# Supplementary material for: Help-seeking in older crime victims: A mixed-methods study in collaboration with the Metropolitan Police Service
Source: PLOS Ment Health. 2024 Aug 8;1(3):e0000082. doi: 10.1371/journal.pmen.0000082 (PMC12798353; doi:10.1371/journal.pmen.0000082)
Supplement: Attachment — Submitted filename: Help Seeking - Response to referees-24.04.2024.docx [file pmen.0000082.s001.docx]

**1. Page 5, Study Aims – Quantitative 1– Should it be “Help seeking” instead of “Health seeking behavior”?**

We agree and have modified this accordingly by change the study aims on p5 from Health-Seeking to Help-Seeking.

**2. Model building in the quantitative analysis appears to be completely data driven (ie based on p values). I am not convinced this is the best approach, even if the paper intends to be descriptive. With this approach, only the strongest associations would survive the selection, and they are usually not surprisingly tautologically. Some demographics, such as education, while may not relate to the outcome directly, often moderate the associations found, given known aged heterogeneity and its social patterning.**

Thank you for raising this. As we were aware that only the strongest associations survive in this approach, we were purposefully cautious by selecting a large *p* value for the univariable testing so that any variable of potential relevance may be included for further testing. Our approach is well suited to research where there is no *a-priori* hypotheses about which variables may be most important. No consensus exists about the best method for selecting the predictor variables, but backward elimination is generally the preferred method [1].

We have therefore acknowledged the potential concerns raised by reviewer 1 and added this in the quantitative methods on p9:

*“Multivariable logistic regression models and backward elimination method with P<0.05 were used to select a final set of variables as the predictors. No consensus exists about the best method for selecting the predictor variables, but backward elimination is generally the preferred method [1]”.*

We understand that there are limitations in selecting variables based on p-values, and variables indirectly associated with the outcome (such as instrumental variable or mediator) may be eliminated based on p-values. However, the aim of the quantitative analysis is to determine predictors of help-seeking behaviours in older crime victims’ of crime, not the causes of help-seeking behaviours among these victims.

We added the following on p 20 in the discussion section to clarify the rationale behind the analysis strategy:

*“The aim was to explore potential predictors of help-seeking behaviours (association) instead of cause of help-seeking behaviours (causality) [23]. However, as the potential predictors were chosen using univariable logistic regression models and p-values, it is possible that variables indirectly associated with help-seeking behaviours may not have been selected”.*

**Do the authors have any specific hypothesis as to how these variables may be related to help seeking?**

There has been very little research in older community crime victims [2],

and prior to our study, we are not aware of any studies of help seeking in older victims of crime.

A number of variables are associated with health outcomes. For example, people from minority groups are often marginalised from receiving care, or as reported in our previous qualitative work [3] older people with psychiatric illness did not feel that being a victim of crime was a legitimate reason to seek help. We therefore considered a range of demographic factors we thought may be associated with help seeking and were also guided by help-seeking research in other victims groups, such as those described in McCart, Smith and Sawyer [4] and Youstin and Siddique [5].

We used a data-driven approach to model building, as it is well suited to studies which do not have *a-priori* hypotheses. We have however added this sentence on p 9.

“*Given the dearth of research determining help-seeking in older victims of community crime, we used a predominantly data-driven approach, although we did consider variables known to be associated with access to health care and help-seeking in other victim groups [8, 12]”*

**3. The barriers and facilitators identified from the interviews are interesting, but not specific to older adults. This is fine, but one may wonder why the paper chose to focus on the older population and what the implications are for older adults.**

Thank you for the feedback and we were pleased the reviewer found the barriers and facilitators interesting.

It may be worth providing the context why our research focussed on an older population and also the implications of the findings.

Our previous work in depression in older adults found that being a victim of crime was often cited as a cause for an older person developing depression and/or anxiety. We became aware that older people may be more vulnerable because of the frailty associated with old age. However, there has been virtually no published work on community crime in this population [2] with most previous work in older victims focussing on elder abuse. Despite rates of depression and anxiety being significantly greater in older victims than may be expected in older people generally, there have been no fully powered trials evaluating how best to manage this population.

We agree that the implications from the qualitative findings may not be specific to older adults. However, as we used inductive analysis, we were guided by the data and reported on what the interviewees considered relevant. Some perspectives may be considered specific to older adults (e.g., physical health concerns, how relationships with doctors have changed over the decades) whilst others may also be relevant to other age groups (e.g., perceptions of NHS wait times). It should not be assumed that the perspectives of older adults only apply to their age group and this wider applicability to other age groups may be considered a strength. Furthermore this approach adheres to guidance from the WHO [6], which advises researchers to consider older adults holistically and move away from viewing ageing only in terms of functional decline.

The referees point is welcomed and we think raises an important consideration about research in older adults. We have therefore added the following paragraph in the discussion on p 24:

*“We used inductive qualitative analysis so were guided by the data and reported what the interviewees considered relevant. Some of the barriers and facilitators may be present in people of all ages, and also associated with cultural and physical factors specific to an older population. The role of GPs is to help with mental health and physical problems. Because of the frailty associated with older age, older people are high utilisers of General Practice services and testing the role of signposting to GPs was considered the first step in deciding how to manage older victims. The recommendations made by older people to consider referral to voluntary and faith organisations may also be applicable to a younger population as part of victim care”*

**Reviewer #2: It is a very interesting paper. However, it is not easy to read the first couple of pages of the paper due to the high number of acronyms but it is manageable. Please explain acronyms like GP since not every non-native reader (as I am) knows that. I googled it but it would be better if you explain it in the first appearance.**

We were pleased reviewer 2 also found the paper interesting.

We appreciate that the use of acronyms can make reading difficult and with the exception of the term VIP (Victim Improvement package), General Practitioner (GP) and commonly used abbreviations for the outcome measures (GAD-2 and PHQ-2) we have removed all the acronyms. A full terminology of the acronyms used and abbreviations have been included the first time they appear in the paper.

We have also clarified the term General Practitioner by

a) Including the equivalent US term (Family Physician) in the abstract.
b) Defining what a General Practitioner is by adding the following sentence on p6.

“A General Practitioner (GP) or family physician is a physician who treats acute and chronic illnesses and provides preventive care and health education to patients of all ages, and who works primarily in a community setting”.

1.	Royston, P., et al., *Prognosis and prognostic research: Developing a prognostic model.* BMJ, 2009. **338**: p. b604.

2.	Satchell, J., et al., *Psychological distress and interventions for older victims of crime: a systematic review.* Trauma, Violence, & Abuse, 2022. **24**(5): p. 3493-3512.

3.	Serfaty, M., et al., *Helping aged victims of crime (the HAVoC study): common crime, older people and mental illness.* Behavioural and Cognitive Psychotherapy, 2016. **44**(2): p. 140-55.

4.	McCart, M.R., D.W. Smith, and G.K. Sawyer, *Help-seeking among victims of crime: A review of the empirical literature.* Journal of Traumatic Stress, 2010. **23**(2): p. 198-206.

5.	Youstin, T.J. and J.A. Siddique, *Psychological Distress, Formal Help-Seeking Behavior, and the Role of Victim Services Among Violent Crime Victims.* Victims & Offenders, 2019. **14**(1): p. 52-74.

6.	World Health Organisation. *WHO's work on the UN Decade of Healthy Ageing (2021–2030): Join the movement on the Decade Platform*. 2022; Available from: <https://www.who.int/initiatives/decade-of-healthy-ageing>.
